# Investigating the pathways from preconception care to preventing maternal, perinatal and child mortality: A scoping review and causal loop diagram

**DOI:** 10.1016/j.pmedr.2023.102274

**Published:** 2023-06-05

**Authors:** Sébastien Poix, Khalifa Elmusharaf

**Affiliations:** a School of Medicine, University of Limerick, Limerick, Ireland; b Applied Health Research, University of Birmingham Dubai, Dubai, United Arab Emirates

**Keywords:** Preconception care, Maternal health, Child health, Causal loop diagram, System thinking, Scoping review

## Abstract

In recent years, there has been a growing recognition that developing preconception care provides an opportunity to significantly reduce maternal and child mortality and morbidity. This involves targeting multiple risk factors through a large array of medical, behavioural and social interventions. In this study, we created a Causal Loop Diagram (CLD) to describe several pathways by which a set of preconception interventions may lead to women's improved health and better pregnancy outcomes. The CLD was informed by a scoping review of *meta*-analyses. It summarises evidence on the outcomes and interventions related to eight preconception risk factors. The authors reviewed literature from two databases (PubMed and Embase) and used the framework developed by Arksey and O'Malley. The CLD includes 29 constructs categorised into five different levels (mortality, causes of death, preconception risk factors, intermediate factors, interventions or policies). The model indicates interconnections between five sub-systems and highlights the role of preventing early and rapidly repeated pregnancies, as well as optimising women's nutritional status in the preconception period. It also shows the prevention of preterm birth as a privileged route for lowering child mortality and morbidity. The CLD demonstrates the potential benefits of strategies that address multiple preconception risk factors simultaneously and can be used as a tool to promote the integration of preconception care into efforts to prevent maternal and child mortality. With further improvements, this model could serve as a basis for future research on the costs and benefits of preconception care.

## Highlights

• A Causal Loop Diagram (CLD) was informed by evidence from 34 *meta*-analyses.
• The CLD uncovers preconception pathways for improving maternal and child health.
• It is key to prevent early/repeated pregnancies and optimise nutritional status.
• The CLD provides a tool for advocating better integration of preconception care.
• Future research should focus on sub-systems and diversify the data sources.

## 1 Introduction

Every day, around 800 women and 6,700 neonates die from preventable pregnancy and childbirth-related complications (World Health Organization, 2019, World Health Organization. Newborn Mortality., 2022). While actions and initiatives conducted since the 60 s led to significant progress in reducing maternal and neonatal mortality, this trend slowed down over the past few decades (Atrash et al., 2006). Also, global progress hides substantial regional inequalities, with the burden of maternal and neonatal mortality being concentrated mainly in low and middle-income countries (World Health Organization, 2019, World Health Organization. Newborn Mortality., 2022). Globally, it is estimated that half of maternal deaths are caused by only three causes: haemorrhage, hypertensive disorders, and sepsis (Say et al., 2014). On its side, neonatal deaths have been prominently attributed to preterm births, birth defects, intrapartum-related complications, and infections (World Health Organization. Newborn Mortality., 2022).

Providing care during pregnancy is crucial to preventing these issues and achieving the best possible pregnancy outcomes. However, it is becoming increasingly apparent that antenatal care only is insufficient since it fails to address health issues and conditions that develop before or in the very first weeks of pregnancy. Sometimes, the first antenatal visit comes too late to identify and address certain risk factors responsible for poor maternal and child health outcomes. There has been clear evidence that health conditions, nutritional deficiencies or exposure to harmful substances in the months before conception can have detrimental effects on foetal growth, maternal and child health (Hemsing et al., 2017, Mason et al., 2014). Ensuring that women enter pregnancy in optimum health is all the more necessary as around half of the women have unintended or unplanned pregnancies globally (Hemsing et al., 2017, Raghuraman and Tuuli, 2021, Korenbrot, 2002). Further, in low-resource settings, access to antenatal care is not always guaranteed, and women can sometimes wait more than four months before having their first visit (Berglund and Lindmark, 2016). As many adverse health outcomes are rooted in the preconception period, extending the continuum of maternal care by addressing pre-pregnancy health risks and health conditions is considered a promising strategy to end preventable maternal and child deaths (World Health Organization, Meeting to develop a global consensus on preconception care to reduce maternal and childhood mortality and morbidity, 2013).

In 2012, the World Health Organization published a global consensus on preconception care (World Health Organization, Meeting to develop a global consensus on preconception care to reduce maternal and childhood mortality and morbidity., 2013). On this occasion, the term was defined as the provision of a range of health interventions to women and couples before conception occurs, whose aim is to improve health status and reduce behaviours, individual and environmental factors that could contribute to poor maternal and child health outcomes. Preconception care involves various health interventions, including health promotion and prevention, risk assessment, and clinical or behavioural interventions. Some key interventions include family planning, micronutrient supplementation, weight management, immunisation, tobacco and alcohol use control, or screening and management of chronic diseases (Hemsing et al., 2017, World Health Organization, Meeting to develop a global consensus on preconception care to reduce maternal and childhood mortality and morbidity, 2013, Goossens et al., 2018).

While robust evidence exists for the effectiveness of certain preconception interventions, the efficacy of others remains uncertain (Lassi et al., 2014, Bhutta, 2011). Micronutrient supplementation before pregnancy has been associated with reduced risks of congenital anomalies (Wilson et al., 2022) and pre-eclampsia (Bodnar et al., 20062006). The effectiveness of folic acid supplementation during the preconception period in reducing neural tube defects has been demonstrated by several studies (De-Regil, 2010). Also, immunisation against measles, mumps and rubella at least three months before pregnancy can prevent congenital rubella syndrome and reduce subsequent risks such as stillbirths, miscarriages, and birth defects (Coonrod et al., 2008). One of the main challenges is precisely measuring the effects of interventions whose effects on maternal or child health are indirect, as is the case with interventions focusing on alcohol and tobacco use, physical activity or violence and domestic abuse, for example.

Researchers and international organisations have called for better integration of preconception care within strategies to prevent maternal and childhood mortality (Atrash et al., 2006, Berglund and Lindmark, 2016, World Health Organization, Meeting to develop a global consensus on preconception care to reduce maternal and childhood mortality and morbidity, 2013, Dorney and Black, 2018). However, only a few countries have developed comprehensive and articulated approaches, such as the United States or the Netherlands (Dorney and Black, 2018). In low and middle-income countries, preconception care interventions have remained scarce or scattered across several public policies. Preconception care services are usually limited to micronutrient supplementation and immunisation, although some more advanced and integrated initiatives have been reported. In Sri Lanka, for example, resources allocated to the maternal care system were leveraged to reach newly married couples with education, screening and clinical services to address domestic violence and manage pre-existing cardiac and neurological conditions (Mason et al., 2014). Several barriers hinder the delivery and uptake of preconception interventions, such as the lack of dedicated guidelines, limited knowledge, time constraints for healthcare providers, and ethical concerns over patients' reproductive autonomy (Dorney and Black, 2018, M’hamdi et al., 2017, Mazza et al., 2013).

The lack of information on the costs and cost-effectiveness of preconception programs has also hindered efforts to develop dedicated policies and guidance. Overall, evidence gathered to date suggests that preconception care interventions are cost-effective (Kotirum et al., 2021). For example, a systematic review (Rodrigues et al., 2021) found that each dollar invested in mandatory folic acid fortification would return a benefit of around $17. A cost-benefit analysis of a preconception care program targeting women with diabetes indicated that for every additional dollar spent on preconception interventions, $1.86 was saved in direct medical costs (Elixhauser et al., 1993). However, evidence is globally limited and fragmented, and further studies are needed to fully involve policymakers and healthcare providers (Grosse et al., 2006). According to a recent systematic review (Kotirum et al., 2021), research priorities should focus on countries with poor pregnancy outcomes and a high burden of maternal or child mortality and morbidity because this is where preconception care is expected to have maximum efficiency. Also, there is a need for more comprehensive economic evaluations that include a broader range of services (Kotirum et al., 2021, Grosse et al., 2006), such as those aiming at preventing unintended pregnancies, reducing violence against women, or addressing unhealthy behaviours.

The aim of the study is to develop a Causal Loop Diagram (CLD) describing the different pathways through which preconception interventions may affect a set of pregnancy outcomes and, in turn, impact maternal, perinatal and child mortality. A CLD is a system thinking tool that describes interactions and feedback mechanisms within a system of interconnected variables (Lin et al., 2020). CLDs are particularly useful for uncovering complex systems' structure and identifying leverage points for initiating positive change. As they allow stakeholders to understand complex problems better, visualise the mechanisms at play and identify opportunities, they are an efficient tool to inform policies and practice (Baugh Littlejohns et al., 2021, US Agency for International Development, 2014). They can also be used to build more advanced quantitative modelling. For these reasons, CLDs have become increasingly popular in public health research (Baugh Littlejohns et al., 2021). In a CLD, the system complexity is visually represented by different elements (variables, arrows, feedback loops). All these elements are used according to certain conventions. A positive relationship between two variables is represented by an arrow with positive polarity ('+'), while a negative relationship is represented by an arrow with negative polarity ('−'). In certain sections of the CLD, linkages between variables can create reinforcing ('R') or balancing ('B') feedback loops. A feedback loop occurs when a change in one variable leads to a change in a second variable that, in turn, affects the initial variable.

The CLD will serve as a tool for understanding the bigger picture and exploring the connections between the many areas covered by the concept of preconception care. Additionally, this study is the first of a series of works that, taken altogether, will investigate the economic costs and benefits of preconception care. In this perspective, understanding the linkages from preconception interventions to maternal, perinatal and child mortality is a starting point for building a more advanced model and developing an investment case methodology for preconception care.

## 2 Materials and methods

We conducted a scoping review of *meta*-analyses to inform the development of the CLD. The review aimed to identify the different constructs to be included in the CLD. The method adopted for this scoping review was informed by the six-stage methodological framework developed by Arksey and O'Malley (Arksey and O'Malley, 2005). The PRISMA-ScR reporting guidelines and checklist (Tricco et al., 2018) were used to ensure consistent and appropriate reporting of the results.

### 2.1 Scope of the CLD

As a first step, a preliminary literature review was conducted to determine the scope of this research and define the research questions. We established a list of preconception risk factors and health outcomes to be included in the CLD. This selection was made after careful consideration and discussion between the authors to ensure they were relevant and representative of preconception care most critical issues.

We included nine preconception risk factors: *Adolescent Pregnancy*, *Short Birth Spacing*, *Pre-Pregnancy Underweight*, *Pre-Pregnancy Overweight*, *Micronutrient Deficiencies*, *Early and Pre-Pregnancy Smoking*, *Early and Pre-Pregnancy Alcohol Use, Vaccine-Preventable Diseases, and Abuse Before Pregnancy*. The notion of preconception risk factor was defined as any condition, behaviour, social or environmental risk factors associated with adverse pregnancy, maternal or perinatal outcomes, whose effects could be eliminated or mitigated through intervention before pregnancy is established. The preconception risk factors were selected based on their frequency of occurrence in the preliminary literature review. Additionally, one of the key considerations was their relevance to a maximum number of women. This ensures that interventions aimed at addressing these risk factors have the potential to impact a significant portion of the population and can lead to improvements in maternal and child health outcomes on a large scale. We did not include already-established medical conditions (i.e. diabetes, hypertension, epilepsy), genetic risks or a history of pregnancy complications.

The authors identified seven health outcomes to be included in the CLD: *Preterm Birth*, *Small for Gestational Age (SGA)*, *Congenital Anomalies*, *Gestational Diabetes Mellitus (GDM)*, *Maternal Hypertensive Disorders*, *Maternal Haemorrhage*, and *Maternal Anaemia*. The selection of health outcomes was made by identifying the most frequently reported outcomes in the preliminary literature review. Also, outcomes that were found to contribute significantly to maternal and neonatal mortality were given particular attention (World Health Organization, 2019, World Health Organization. Newborn Mortality., 2022, Say et al., 2014).

### 2.2 Identifying the research questions

In coherence with the scope of the CLD and the considerations listed above, two research questions were identified by the research team: (1) to what maternal, perinatal, or child health outcomes the selected preconception risk factors are commonly associated, and (2) what preconception interventions or policies positively influence these risk factors?

### 2.3 Identifying relevant studies

The electronic databases PubMed and Embase were searched in August and September 2022. Multiple search strategies were used to identify studies related to outcomes and interventions. The combinations of keywords used are reported as Supplemental Material (Supplemental Material n°1). Search terms were limited to titles and abstracts, and only studies written in English were included. Considering the scope of this study and the volume of associated literature, we decided to focus on results produced by *meta*-analyses. To make sure we obtained the most up-to-date evidence, *meta*-analyses published before 2002 were excluded. To be included, *meta*-analyses had to study one of the selected preconception risk factors and demonstrate an association with at least an outcome of interest and/or a preconception intervention or policy.

### 2.4 Charting the data

The first author (SP) screened all the titles and abstracts, applying inclusion criteria defined by the research team. SP assessed the studies eligible for full-text review, and the second author (KH) made the final decision in case of doubt. The characteristics of all included studies were extracted and entered into an excel table containing the following categories: *Title*, *Year*, *Main Author*, *Intervention or Risk Factor*, *Outcome*, *and Results*.

### 2.5 Collating, summarising and reporting the results using a CLD

The data extracted from the 34 included studies were reviewed to identify the adverse maternal, perinatal and child health outcomes associated with preconception risk factors and the interventions or policies affecting these risk factors. The authors created a spreadsheet including, for each *meta*-analysis, the variables identified and the different connections linking them. For better clarity, similar variables extracted from different studies were grouped into the same variable label. In the CLD, we used grey dash lines to indicate potential linkages between constructs. These lines show connections that were not demonstrated by the *meta*-analyses but which can be reasonably assumed based on the existing literature. The inclusion of these connections in the final CLD was made after careful consideration and discussion to ensure that they were pertinent to the model and contributed to its overall value. The CLD was created with the software Vensim PLE 9.1.1. An iterative approach was used after the two authors had agreed on the variables and linkages to be included.

## 3 Results

### 3.1 Study selection

**Fig. 1:**
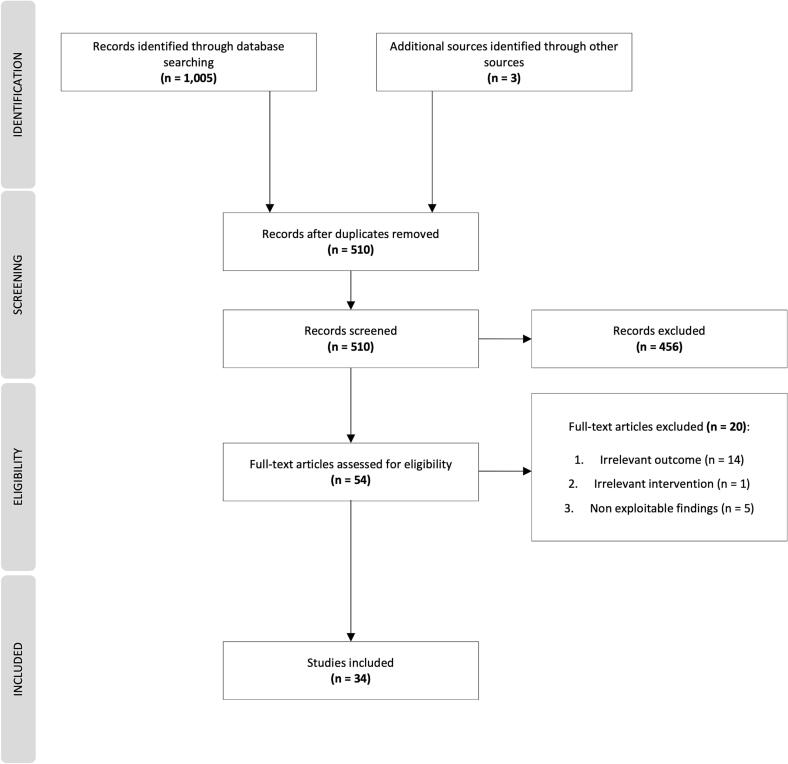
**PRISMA Flow Diagram** Description: The flow diagram illustrates the different stages of the literature search.

The systematic search yielded 1,008 records, including 1,005 records from electronic databases and 3 from hand-searching. 510 studies remained after removing the duplicates. After screening the titles and abstracts, 456 studies were excluded as they did not fulfil the inclusion criteria. In total, 54 studies were eligible for full-text review. 20 were excluded because the outcomes or interventions were irrelevant or the results were non-exploitable. Finally, 34 *meta*-analyses were included in the scoping review (Fig. 1).

### 3.2 Characteristics of the studies

Table 1 shows the studies included in this review, the relevant outcomes and associated results. The last column lists the different variables and connections used to develop the CLD. In total, we used data extracted from 34 *meta*-analyses. Nine studies included a variable related to adolescent pregnancy (Chin et al., 2012, Dean et al., 2014, Grønvik et al., 2018, Harden et al., 2009, Kozuki et al., 2013, Laelago et al., 2020, Lassi et al., 2020, Marvin-Dowle and Soltani, 2020, Mason-Jones et al., 2016), five to short birth spacing (Conde-Agudelo et al., 2006, Damtie et al., 2021, Dean et al., 2014, Kozuki et al., 2013, Laelago et al., 2020), seven to micronutrient deficiencies (De-Regil et al., 2015, Dean et al., 2014, Imdad et al., 2011, Keats et al., 2019, Lassi et al., 2020, Li et al., 2019, Zhang et al., 2017), five to pre-pregnancy underweight (Dean et al., 2014, Liu et al., 2016, Liu et al., 2019, Vats et al., 2021, Yu et al., 2013), ten to pre-pregnancy overweight and obesity (Dean et al., 2014, He et al., 2020, Liu et al., 2016, Liu et al., 2019, Najafi et al., 2019, Putra et al., 2022, Ulhaq et al., 2021, Vats et al., 2021, Yang et al., 2021, Zhang et al., 2021), two to pre-pregnancy smoking (Lassi et al., 2014, Zhang et al., 2021); one to vaccine-preventable diseases (Ye et al., 2019), and one to abuse before pregnancy (Nesari et al., 2018). Twenty-one studies analysed the effects of preconception risk factors on maternal or perinatal outcomes (Conde-Agudelo et al., 2006, Dean et al., 2014, Dean et al., 2014, Grønvik et al., 2018, He et al., 2020, Kozuki et al., 2013, Kozuki et al., 2013, Laelago et al., 2020, Lassi et al., 2014, Liu et al., 2016, Liu et al., 2019, Marvin-Dowle and Soltani, 2020, Najafi et al., 2019, Nesari et al., 2018, Putra et al., 2022, Ulhaq et al., 2021, Vats et al., 2021, Yang et al., 2021, Ye et al., 2019, Yu et al., 2013, Zhang et al., 2021), and fifteen studies analysed the effects of interventions or policies on the selected preconception risk factors or outcomes (Aune et al., 2016, Aune et al., 2014, Chin et al., 2012, Damtie et al., 2021, De-Regil et al., 2015, Dean et al., 2014, Dean et al., 2014, Harden et al., 2009, Imdad et al., 2011, Keats et al., 2019, Lassi et al., 2020, Li et al., 2019, Mason-Jones et al., 2016, Mijatovic-Vukas et al., 2018, Zhang et al., 2017). Among the studies focusing on maternal or perinatal outcomes, thirteen reported outcome measures on preterm birth (Conde-Agudelo et al., 2006, Dean et al., 2014, Dean et al., 2014, Grønvik et al., 2018, Kozuki et al., 2013, Kozuki et al., 2013, Laelago et al., 2020, Li et al., 2019, Liu et al., 2016, Liu et al., 2019, Marvin-Dowle and Soltani, 2020, Nesari et al., 2018, Vats et al., 2021), ten on small for gestational age (Conde-Agudelo et al., 2006, Dean et al., 2014, Dean et al., 2014, Kozuki et al., 2013, Kozuki et al., 2013, Liu et al., 2016, Liu et al., 2019, Vats et al., 2021, Yu et al., 2013, Zhang et al., 2017), eight on congenital anomalies (De-Regil et al., 2015, Dean et al., 2014, Imdad et al., 2011, Keats et al., 2019, Lassi et al., 2014, Lassi et al., 2020, Putra et al., 2022, Ye et al., 2019), three on stillbirth (Dean et al., 2014, Liu et al., 2016, Vats et al., 2021), three on anaemia (Dean et al., 2014, Keats et al., 2019, Lassi et al., 2020), six on maternal hypertensive disorders (Aune et al., 2014, Dean et al., 2014, Grønvik et al., 2018, He et al., 2020, Ulhaq et al., 2021, Vats et al., 2021), six on gestational diabetes mellitus (Aune et al., 2016, Dean et al., 2014, Mijatovic-Vukas et al., 2018, Najafi et al., 2019, Vats et al., 2021, Zhang et al., 2021), and one on maternal haemorrhage (Vats et al., 2021). Fifteen reviewed studies reported the effects of interventions or policies on the selected preconception risk factors or outcomes, including interventions designed to prevent adolescent and rapid repeat pregnancies (Chin et al., 2012, Dean et al., 2014, Harden et al., 2009, Lassi et al., 2020, Mason-Jones et al., 2016), preconception folic acid supplementation (De-Regil et al., 2015, Dean et al., 2014, Lassi et al., 2020, Li et al., 2019, Zhang et al., 2017), preconception iron supplementation (Lassi et al., 2020), and food fortification (Imdad et al., 2011, Keats et al., 2019). In addition, a few studies explored the benefits of pre-pregnancy physical activity (Aune et al., 2016, Aune et al., 2014, Mijatovic-Vukas et al., 2018), contraception use (Chin et al., 2012), and optimal breastfeeding duration (Damtie et al., 2021) in relation to one or more selected preconception risk factors.

**Table 1:** Characteristics of the included studies and variables used in the CLD.

**Study**	**Title**	**Outcome(s)**	**Findings**	**Variables and Linkages**
(30) Aune et al. (2016)	Physical activity and the risk of gestational diabetes mellitus: a systematic review and dose–response *meta*-analysis of epidemiological studies	Gestational diabetes mellitus	Higher total physical activity (RR = 0.62, 95% CI: 0.41–0.94) and leisure-time physical activity (RR = 0.78, 95% CI: 0.61–1.00) before pregnancy were associated with reduced risks of gestational diabetes mellitus	**Pre-Pregnancy Physical Activity -> (-) Gestational Diabetes Mellitus**
(31) Aune et al. (2014)	Physical activity and the risk of preeclampsia: a systematic review and *meta*-analysis	Preeclampsia	Higher physical activity before pregnancy was associated with reduced risks of preeclampsia (RR = 0.65, 95% CI: 0.47–0.89)	**Pre-Pregnancy Physical Activity -> (-) Maternal Hypertensive Disorders**
(32) Chin et al. (2012)	The effectiveness of group-based comprehensive risk-reduction and abstinence education interventions to prevent or reduce the risk of adolescent pregnancy, human immunodeficiency virus, and sexually transmitted infections: two systematic reviews for the Guide to Community Preventive Services	Protective sexual behaviours, unprotected sexual activity	Comprehensive risk-education interventions were associated with reduced unprotected sexual activity (OR = 0.70, 95% CI: 0.60–0.82) and increased protective sexual behaviours (OR = 1.39, 95% CI: 1.19–1.62)	**Risk Reduction Intervention -> (-) High-Risk Sexual Behaviours** **Risk Reduction Intervention -> (+) Contraception Use**
(33) Conde-Agudelo et al. (2006)	Birth spacing and risk of adverse perinatal outcomes: a *meta*-analysis	Preterm birth, small for gestational age	Short birth interval (<18 months) was associated with increased risks of preterm birth (OR = 1.92, 95% CI: 1.80–3.04) and small for gestational age (OR = 1.52, 95% CI: 1.40–1.64)	**Short Birth Spacing -> (+) Preterm Birth** **Short Birth Spacing -> (+) SGA**
(34) Damtie et al. (2021)	Short birth spacing and its association with maternal educational status, contraceptive use, and duration of breastfeeding in Ethiopia. A systematic review and *meta*-analysis	Short birth spacing	Women who practised breastfeeding for less than 24 months (OR = 16.9, 95% CI: 2.69–106.47) and women who had never used contraception (OR = 3.87, 95% CI: 2.29–6.53) had increased risks of short birth spacing	**Breastfeeding Duration -> (-) Short Birth Spacing** **Contraception Use -> (-) Short Birth Spacing**
(35) De-Regil et al. (2015)	Effects and safety of periconceptional folate supplementation for preventing birth defects	Neural tube defects	Folic acid supplementation before pregnancy was associated with reduced risks of neural tube defects (RR = 0.31, 95% CI: 0.16–0.60)	**Preconception Folic Acid Supplementation -> (-) Congenital Anomalies**
(36) Dean et al. (2014)	Preconception care: Promoting reproductive planning	Adolescent pregnancy, rapid repeat adolescent pregnancy, maternal anaemia, preterm birth, small for gestational age, stillbirth	Comprehensive interventions were associated with reduced risks of adolescent pregnancy (OR = 0.85, 95% CI: 0.74–0.98) and rapid repeat adolescent pregnancy (OR = 0.63, 95% CI: 0.49–0.82). Short birth interval was associated with increased risks of preterm birth (OR = 1.45, 95% CI: Not Reported), small for gestational age (OR = 1.17, 95% CI: Not Reported), maternal anaemia (OR = 1.32, 95% CI: Not Reported), and stillbirth (OR = 1.42, 95% CI: 1.09–1.86)	**Risk Reduction Intervention -> (-) Adolescent Pregnancy** **Risk Reduction Intervention -> (-) Short Birth Spacing** **Short Birth Spacing -> (+) Preterm Birth** **Short Birth Spacing -> (+) SGA** **Short Birth Spacing -> (+) Maternal Anaemia** **Short Birth Spacing -> (+) Stillbirth**
(37) Dean et al. (2014)	Preconception care: nutritional risks and interventions	Preterm birth, small for gestational age, preeclampsia, gestational diabetes mellitus, birth defects, neural tube defects, congenital heart defects, congenital anomalies	Pre-pregnancy underweight was associated with increased risks of preterm birth (RR = 1.32, 95% CI: 1.22–1.43) and small for gestational age (RR = 1.64, 95% CI: 1.22–2.21). Pre-pregnancy overweight was associated with increased risks of preeclampsia (OR = 2.28, 95% CI: 2.04–2.55), gestational diabetes mellitus (OR = 1.91, 95% CI: 1.58–2.32), and birth defects (OR = 1.15, 95% CI: 1.07–1.24). Folic acid supplementation was associated with reduced risks of neural tube defects (RR = 0.31, 95% CI: 0.14–0.66) and congenital heart defects (OR = 0.58, 95% CI: 0.42–0.79).	**Pre-Pregnancy Underweight -> (+) Preterm Birth** **Pre-Pregnancy Underweight -> (+) SGA** **Pre-Pregnancy Overweight and Obesity -> (+) Maternal Hypertensive Disorders** **Pre-Pregnancy Overweight and Obesity -> (+) Gestational Diabetes Mellitus** **Pre-Pregnancy Overweight and Obesity -> (+) Congenital Anomalies** **Preconception Folic Acid Supplementation -> (-) Congenital Anomalies**
(38) Grønvik et al. (2018)	Complications associated with adolescent childbearing in Sub-Saharan Africa: A systematic literature review and *meta*-analysis	Preterm birth, preeclampsia/eclampsia	Adolescent pregnancy was associated with increased risks of preterm birth (AOR = 1.75, 95% CI: 1.18–2.61) and preeclampsia (AOR = 3.52, 95% CI: 2.26–5.48)	**Adolescent Pregnancy -> (+) Preterm Birth** **Adolescent Pregnancy -> (+) Maternal Hypertensive Disorders**
(39) Harden et al. (2009)	Teenage pregnancy and social disadvantage: systematic review integrating controlled trials and qualitative studies.	Adolescent pregnancy	Youth development programs were associated with reduced adolescent pregnancy rates (RR = 0.55, 95% CI: 0.40–0.76)	**Youth Development Program -> (-) Adolescent Pregnancy**
(40) He et al. (2020)	Maternal prepregnancy overweight and obesity and the risk of preeclampsia: A *meta*-analysis of cohort studies	Preeclampsia	Pre-pregnancy overweight (AOR = 1.71, 95% CI: 1.52–1.91) and pre-pregnancy obesity (AOR = 2.48, 95% CI: 2.05–2.90) were associated with increased risks of preeclampsia	**Pre-Pregnancy Overweight or Obesity -> (+) Maternal Hypertensive Disorders**
(41) Imdad et al. (2011)	The effect of folic acid, protein energy and multiple micronutrient supplements in pregnancy on stillbirths	Neural tube defects	Folic acid fortification (RR = 0.59, 95% CI: 0.52–0.68) and supplementation (RR = 0.38, 95% CI: 0.29–0.51) were associated with reduced risks of neural tube defects	**Folic Acid Fortification -> (-) Congenital Anomalies** **Folic Acid Supplementation -> (-) Congenital Anomalies**
(42) Keats et al. (2019)	Improved micronutrient status and health outcomes in low- and middle-income countries following large-scale fortification: evidence from a systematic review and *meta*-analysis	Anaemia prevalence in women of reproductive age, neural tube defects	Large-scale iron fortification was associated with reduced anaemia prevalence in women of reproductive age (RR = 0.66 95% CI: 0.58–0.76). Folic acid fortification was associated with reduced risks of neural tube defects (OR = 0.59, 95% CI: 0.49–0.70)	**Food Fortification -> (-) Anaemia** **Food Fortification -> (-) Congenital Anomalies**
(43) Kozuki et al. (2013)	The associations of birth intervals with small-for-gestational age, preterm, and neonatal and infant mortality: a *meta*-analysis	Small for gestational age, preterm birth	Birth interval shorter than 18 months was associated with increased risks of small for gestational age (AOR = 1.51, 95% CI: 1.31–1.75) and preterm birth (AOR = 1.58, 95% CI: 1.19–2.10)	**Short Birth Spacing -> (+) SGA** **Short Birth Spacing -> (+) Preterm Birth**
(44) Kozuki et al. (2013)	The associations of parity and maternal age with small-for-gestational-age, preterm, and neonatal and infant mortality: a *meta*-analysis	Small for gestational age, preterm birth	Nulliparous aged less than 18 had increased risks of small for gestational age (AOR = 1.80, 95% CI: 1.62–2.01) and preterm birth (AOR = 1.52, 95% CI: 1.40–1.66)	**Adolescent Pregnancy -> (+) SGA** **Adolescent Pregnancy -> (+) Preterm Birth**
(45) Laelago et al. (2020)	Determinants of preterm birth among mothers who gave birth in East Africa: systematic review and *meta*-analysis	Preterm birth	Age less than 20 (AOR = 1.76, 95% CI: 1.33–2.32), birth interval less than 24 months (AOR = 2.03, 95% CI: 1.57–2.62), pregnancy-induced hypertension (AOR = 3.14, 95% CI: 2.60–4.65), and maternal anaemia (AOR = 4.58, 95% CI: 2.63–7.96) were associated with increased risks of preterm birth	**Adolescent Pregnancy -> (+) Preterm Birth** **Short Birth Spacing -> (+) Preterm Birth** **Maternal Hypertensive Disorders -> (+) Preterm Birth** **Maternal Anaemia -> (+) Preterm Birth**
(46) Lassi et al. (2014)	Preconception care: Caffeine, smoking, alcohol, drugs and other environmental chemical/radiation exposure	Congenital heart defects	Periconception smoking was associated with increased risks of congenital heart defects (OR = 2.80, 95% CI: 1.76–4.47)	**Pre-Pregnancy Smoking -> (+) Congenital Anomalies**
(47) Lassi et al. (2020)	Effects of Preconception Care and Periconception Interventions on Maternal Nutritional Status and Birth Outcomes in Low- and Middle-Income Countries: A Systematic Review	Neural tube defects, maternal anaemia, contraception use	Periconceptional folic acid supplementation was associated with reduced risks of neural tube defects (RR = 0.53, 95% CI: 0.41–0.67). Periconceptional iron-folic acid supplementation was associated with reduced risks of anaemia (RR = 0.66, 95% CI: 0.53–0.81). Educational interventions on contraception use were associated with increased contraception use (RR = 4.69, 95% CI: 3.22–6.83)	**Folic Acid Supplementation -> (-) Congenital Anomalies** **Iron Supplementation -> (-) Anaemia** **Risk Reduction Intervention -> (+) Contraception Use**
(48) Li et al. (2019)	Folic Acid and Risk of Preterm Birth: A Meta-Analysis	Preterm birth	Folic acid supplementation before conception was associated with reduced risks of preterm birth (OR = 0.87, 95% CI: 0.84–0.91)	**Folic Acid Supplementation -> (-) Preterm Birth**
(49) Liu et al. (2016)	Association between perinatal outcomes and maternal pre-pregnancy body mass index	Stillbirth, small for gestational age, preterm birth	Pre-pregnancy overweight (OR = 1.27, 95% CI: 1.18–1.36) and obesity (OR = 1.81, 95% CI: 1.69–1.93) were associated with increased risks of stillbirth. Pre-pregnancy underweight was associated with increased risks of small for gestational age (OR = 1.67, 95% CI: 1.49–1.87) and preterm birth (OR = 1.30, 95% CI: 1.13–1.49).	**Pre-Pregnancy Overweight or Obesity -> (+) Stillbirth** **Pre-Pregnancy Underweight -> (+) Preterm Birth** **Pre-Pregnancy Underweight -> (+) SGA**
(50) Liu et al. (2019)	Maternal body mass index and risk of neonatal adverse outcomes in China: a systematic review and *meta*-analysis	Preterm birth, small for gestational age	Pre-pregnancy underweight was associated with increased risks of small for gestational age (OR = 1.75, 95% CI: 1.51–2.02). Pre-pregnancy overweight or obesity was associated with increased risks of preterm birth (OR = 1.38, 95% CI: 1.25–1.52)	**Pre-Pregnancy Underweight -> (+) SGA** **Pre-Pregnancy Overweight or Obesity -> (+) Preterm Birth**
(51) Marvin-Dowle and Soltani (2020)	A comparison of neonatal outcomes between adolescent and adult mothers in developed countries: A systematic review and *meta*-analysis	Preterm birth	Adolescent pregnancy was associated with increased risks of preterm birth (OR = 1.23, 95% CI: 1.09–1.38)	**Adolescent Pregnancy -> (+) Preterm Birth**
(52) Mason-Jones et al. (2016)	School-based interventions for preventing HIV, sexually transmitted infections, and pregnancy in adolescents	Adolescent pregnancy	Pregnancy was reduced in those who received material or monetary incentive-based programmes (RR = 0.76, 95% CI: 0.58 to 0.99)	**Incentive-Based Programme -> (-) Adolescent Pregnancy**
(53) Mijatovic-Vukas et al. (2018)	Associations of Diet and Physical Activity with Risk for Gestational Diabetes Mellitus: A Systematic Review and Meta-Analysis	Gestational diabetes mellitus	Physical activity during the pre-pregnancy period was associated with reduced risks of gestational diabetes mellitus (OR = 0.70, 95% CI: 0.57–0.85)	**Physical Activity Before Pregnancy -> (-) Gestational Diabetes Mellitus**
(54) Najafi et al. (2019)	The effect of prepregnancy body mass index on the risk of gestational diabetes mellitus: A systematic review and dose–response *meta*-analysis	Gestational diabetes mellitus	Pre-pregnancy overweight (AOR = 2.01, 95% CI: 1.75–2.26) and obese (AOR = 3.98, 95% CI: 3.42–4.53) were associated with reduced risks of gestational diabetes mellitus	**Pre-Pregnancy Overweight or Obesity -> (+) Gestational Diabetes Mellitus**
(55) Nesari et al. (2018)	Does a maternal history of abuse before pregnancy affect pregnancy outcomes? A systematic review with *meta*-analysis	Preterm birth	Maternal abuse occurring within 12 months before pregnancy was associated with increased risks of preterm birth (OR = 1.28, 95% CI: 1.09–1.49)	**Abuse Before Pregnancy –> (+) Preterm Birth**
(56) Putra et al. (2022)	Pre-pregnancy obesity and the risk of peripartum cardiomyopathy: A systematic review and *meta*-analysis	Peripartum cardiomyopathy	Pre-pregnancy obesity was associated with increased risks of peripartum cardiomyopathy (OR = 1.79, 95% CI: 1.16–2.76)	**Pre-Pregnancy Overweight or Obesity -> (+) Congenital Anomalies**
(57) Ulhaq et al. (2021)	Association between pre-pregnancy body mass index and gestational weight gain and the risk of preeclampsia: A systematic review and *meta*-analysis	Preeclampsia	Pre-pregnancy overweight (OR = 2.15, 95% CI: 1.36–3.40) and obesity (OR = 2.86, 95% CI: 1.76–4.65) were associated with increased risks of preeclampsia.	**Pre-Pregnancy Overweight or Obesity -> (+) Maternal Hypertensive Disorders**
(58) Vats et al. (2021)	Impact of maternal pre-pregnancy body mass index on maternal, fetal and neonatal adverse outcomes in the worldwide populations: A systematic review and *meta*-analysis	Gestational diabetes mellitus, gestational hypertension, preeclampsia, postpartum haemorrhage, preterm birth, small for gestational age, stillbirth, neonatal asphyxia	Pre-pregnancy underweight was associated with increased risks of preterm birth (OR = 1.22, 95% CI: 1.16–1.27) and small for gestational age (OR = 1.55, 95% CI: 1.49–1.62). Pre-pregnancy overweight was associated with increased risks of gestational diabetes mellitus (OR = 2.10, 95% CI: 1.89–2.33), gestational hypertension (OR = 2.24, 95% CI: 1.94–2.59), preeclampsia (OR = 1.89, 95% CI: 1.74–2.05), postpartum haemorrhage (OR = 1.18, 95% CI: 1.11–1.26), preterm birth (OR = 1.04, 95% CI: 1.01–1.07), and stillbirth (OR = 1.23, 95% CI: 1.12–1.36). Pre-pregnancy obesity was associated with increased risks of gestational diabetes mellitus (OR = 4.10, 95% CI: 3.50–4.80), gestational hypertension (OR = 4.77, 95% CI: 3.88–5.85), preeclampsia (OR = 3.57, 95% CI: 3.29–3.87), postpartum haemorrhage (OR = 1.38, 95% CI: 1.25–1.54), preterm birth (OR = 1.17, 95% CI: 1.13–1.21), and stillbirth (OR = 1.54, 95% CI: 1.35–1.75).	**Pre-Pregnancy Underweight -> (+) Preterm Birth** **Pre-Pregnancy Underweight -> (+) SGA** **Pre-Pregnancy Overweight or Obesity -> (+) Gestational Diabetes Mellitus** **Pre-Pregnancy Overweight or Obesity -> (+) Maternal Hypertensive Disorders** **Pre-Pregnancy Overweight or Obesity -> (+) Maternal Haemorrhage** **Pre-Pregnancy Overweight or Obesity -> (+) Preterm Birth** **Pre-Pregnancy Overweight or Obesity -> (+) Stillbirth**
(59) Yang et al. (2021)	The effect of prepregnancy body mass index on maternal micronutrient status: a *meta*-analysis	Folate deficiency during pregnancy	Pre-pregnancy obesity (OR = 1.69, 95% CI: 1.32–2.16) and pre-pregnancy overweight (OR = 1.57, 95% CI: 1.05–2.34) were associated with increased risks of folate deficiency during pregnancy	**Pre-Pregnancy Overweight or Obesity -> (+) Folic Acid Deficiency**
(60) Ye et al. (2019)	Maternal Viral Infection and Risk of Fetal Congenital Heart Diseases: A Meta-Analysis of Observational Studies	Congenital heart diseases	Infants born from mothers infected with the rubella virus in early pregnancy had higher risks of developing congenital heart diseases (OR = 3.54, 95% CI: 1.75–7.15)	**Vaccine-Preventable Diseases -> (+) Congenital Anomalies**
(61) Yu et al. (2013)	Pre-pregnancy body mass index in relation to infant birth weight and offspring overweight/obesity: a systematic review and *meta*-analysis	Small for gestational age	Pre-pregnancy underweight was associated with increased risks of small for gestational age (OR = 1.81, 95% CI: 1.76–1.87).	**Pre-Pregnancy Underweight -> (+) SGA**
(62) Zhang et al. (2017)	Effect of folic acid supplementation on preterm delivery and small for gestational age births: A systematic review and *meta*-analysis	Small for gestational age	Folic acid supplementation before conception was associated with reduced risks of small for gestational age (RR = 0.70, 95% CI: 0.57–0.85)	**Folic Acid Supplementation -> (-) SGA**
(63) Zhang et al. (2021)	Factors Associated with Gestational Diabetes Mellitus: A Meta-Analysis	Gestational diabetes mellitus	Pre-pregnancy smoking (OR = 2.32, 95% CI: 1.36–3.97) and pre-pregnancy overweight or obesity (OR = 2.64, 95% CI: 1.56–4.45) were associated with increased risks of gestational diabetes mellitus	**Pre-Pregnancy Smoking -> (+) Gestational Diabetes Mellitus** Pre-Pregnancy Overweight or Obesity -> (+) Gestational Diabetes Mellitus

### 3.3 Causal Loop Diagram

**Fig. 2:**
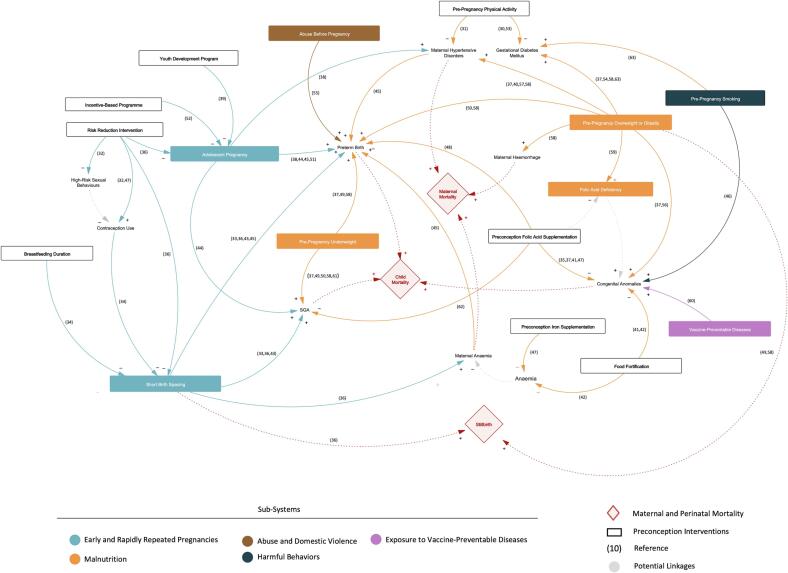
**Causal Loop Diagram (CLD) of preconception care and its contribution to reducing maternal and perinatal mortality [COLOR]** Description: The CLD shows the connections between the different constructs that emerged from the data collection and analysis phase. This visual tool illustrates how a set of preconception interventions can result in improved pregnancy outcomes and ultimately influences maternal and child mortality.

The CLD (Fig. 2) offers a visual representation of the different pathways by which preconception interventions may contribute to lowering maternal, perinatal and child mortality. It includes 29 constructs distributed over five levels: (1) stillbirth, maternal and child mortality; (2) causes of death; (3) preconception risk factors, (4) intermediate mechanisms; (5) preconception interventions or policies. The CLD is also built around five sub-systems identifiable thanks to a colour code: preventing early and rapidly repeated pregnancies (light blue); optimising women's nutritional status (orange); promoting a healthy lifestyle (dark blue); protecting women from the risks of exposure to vaccine-preventable diseases (light purple); preventing domestic violence and abuse (brown).

#### 3.3.1 Neonatal mortality

In the CLD, three health outcomes were linked to neonatal mortality: preterm birth, small-for-gestational-age, and congenital anomalies.

Several *meta*-studies found that adolescent pregnancies and pregnancies following a short birth interval had higher risks of preterm births. The literature also identified pre-pregnancy weight as a risk factor for preterm birth, with three *meta*-analyses demonstrating an association with pre-pregnancy underweight and two with pre-pregnancy overweight and obesity. Furthermore, one study found that women who experienced abuse in the 12 months preceding pregnancy were more likely to give birth to a premature baby. The risks of delivering a small-for-gestational-age baby were found to be higher in adolescent mothers and pregnancies following a short interpregnancy interval. In terms of pre-pregnancy weight, five *meta*-studies demonstrated that underweight mothers had higher risks of delivering a small-for-gestational-age baby. Regarding the risks of congenital anomalies, women who were overweight and obese before pregnancy were more likely to give birth to a baby with orofacial clefts, neural tube defects, and congenital heart defects. The risks of congenital heart defects were also found to be higher in women who smoked before and in early pregnancy. Finally, maternal exposure to the rubella virus in early pregnancy was associated with higher risks of congenital heart diseases in newborns.

The CLD shows that several interventions can mitigate the effects of preconception risk factors leading to neonatal mortality. A few studies suggest that the provision of sexual risk-reduction interventions may play a role in preventing early and rapidly repeated pregnancies, notably through an increase in contraception use and a decrease in high-risk sex behaviours. However, it must be noted that the construct of risk-reduction interventions includes related but distinct approaches (target group, intervention content). Another study shows the positive effects of interventions based on economic and/or material incentives, especially for adolescent women from deprived families or communities. In addition, one *meta*-analysis demonstrates that increased breastfeeding duration was associated with a lower risk of short birth intervals, indicating potential benefits of breastfeeding promotion. The literature also highlights the importance of improving women's nutritional status before pregnancy, which includes achieving optimal weight and addressing micronutrient deficiencies. On this last point, several *meta*-analyses underly the role of preconception supplementation and/or large-scale food fortification in preventing micronutrient deficiencies and reducing the risks of associated events or conditions, such as small for gestational age, preterm birth, congenital anomalies or maternal anaemia. The protective effect of preconception folic acid supplementation on the occurrence of neural tube defects is particularly well identified, with four *meta*-analyses pointing to the same conclusion.

#### 3.3.2 Maternal mortality

Maternal mortality was directly linked to three health outcomes: maternal hypertensive disorders, maternal haemorrhage, and maternal anaemia.

In relation to maternal hypertensive disorders, a *meta*-analyse demonstrated that adolescent pregnancies were at higher risk of pre-eclampsia/eclampsia. Mothers who were overweight or obese before pregnancy also carried a higher risk of pre-eclampsia and gestational hypertension. These associations were supported by five *meta*-studies. Regarding maternal haemorrhage, one *meta*-analysis showed higher risks of postpartum haemorrhage in mothers with pre-pregnancy overweight or obesity. Finally, increased risks of maternal anaemia were found in pregnancies following a short interpregnancy interval.

Interventions addressing short birth spacing, such as sexual risk-reduction interventions, contraceptive promotion and provision, and breastfeeding promotion, may reduce the risks of maternal anaemia, ultimately leading to lowering maternal mortality. Another pathway is the promotion of physical activity in the preconception period, which has been negatively associated with pre-eclampsia. Pre-pregnancy physical activity was also associated with decreased risks of gestational diabetes mellitus, but this outcome was not directly or indirectly connected to maternal mortality in the CLD.

#### 3.3.3 Stillbirth

In the CLD, stillbirths were directly linked with two preconception risk factors: short birth spacing and pre-pregnancy overweight and obesity.

Interventions that address short birth spacing, such as sexual risk-reduction interventions, contraceptive promotion and provision, or breastfeeding promotion, may contribute to preventing stillbirths.

## 4 Discussion

This study aimed to investigate the pathways by which preconception care can contribute to reducing maternal and child mortality. After conducting a scoping review of the literature, we synthesised our results in the form of a CLD. We created a visual tool to help better understand and visualise how preconception risk factors may influence several maternal, child, and pregnancy outcomes and how intervening before pregnancy may eliminate or mitigate these risks.

The model suggests that effective provision of preconception care requires a comprehensive and multisectoral approach, combining behavioural lifestyle interventions, such as promoting improvement in diet, weight and physical activity, family planning, screening programmes, risk reduction interventions, as well as strategies addressing socioeconomic factors. Though limited in scope, the interventions we identified are globally coherent with the study conducted by Lassi et al. (Lassi et al., 2014). They are also partly aligned with preconception interventions identified by the World Health Organization. In the Global Consensus on Preconception Care to Reduce Maternal and Childhood Mortality and Morbidity, recommended interventions do not limit to preconception risk factors included in this study but also address genetic disorders, environmental risks, sexually transmitted infections, infertility and subfertility, female genital mutilation, mental health disorders and psychoactive substance use (World Health Organization, Meeting to develop a global consensus on preconception care to reduce maternal and childhood mortality and morbidity, 2013).

In the CLD, the constructs with the most arrows leaving from them were pre-pregnancy overweight and obesity (n = 7), short birth spacing (n = 4), and adolescent pregnancy (n = 3), indicating that these three risk factors concentrate the most evidence from *meta*-studies. On the contrary, the number of *meta*-analyses investigating the impact of pre-pregnancy smoking, domestic violence and abuse before pregnancy, and exposure to vaccine-preventable diseases is more limited. This imbalance may be due to the fact that pre-pregnancy weight, maternal age and birth intervals can be easily accessed through medical records or health surveys, making studies on these topics easier to conduct. Some risk factors can also present practical challenges in terms of study design. For example, because it may take a few weeks for women to become aware of their pregnancy, it is difficult to accurately separate the effects of smoking prior to pregnancy from the effects of smoking in early pregnancy. 

The health outcomes with the most arrows pointing to them were preterm birth (n = 8), followed by congenital anomalies (n = 5) and small for gestational age (n = 4). The accumulation of evidence regarding the risk of preterm birth is of particular interest since prematurity-related complications remain the leading cause of mortality in children under five (Cao et al., 2022, Walani, 2020). Most of the impact of preconception care on child mortality could be generated through this pathway. Another route relates to lowering the occurrence of birth defects, with an emphasis on preventing neural tube defects through folic acid supplementation or large-scale fortification (Flores, 2014). If controlling folate deficiency is a well-recognised strategy, the benefits of initiating supplementation in other nutrients before pregnancy are not fully established. For example, iron fortification or supplementation in the preconception period may result in greater iron stores during pregnancy, but it is not clear whether such interventions protect women from maternal anaemia and translate, in turn, into improved pregnancy outcomes. The provision of education and advice is often considered a key component of preconception care that could support changes in diet and physical activity, nutritional supplement use or smoking cessation. However, the available evidence has not clearly demonstrated that pre-pregnancy counselling translated into behavioural change and improved health and pregnancy outcomes (Whitworth and Dowswell, 2009).

## 5 Limitations

The final version of the CLD reflects the authors' interpretation of the available data. Thus, it is not an exhaustive model, and it must rather be considered a starting point from which more complex and detailed systems can be modelled. There are other elements and pathways that should be considered when examining how preconception care may reduce maternal and child mortality, such as the role of interventions aiming at reducing environmental risks, genetic risks, or mental health disorders before pregnancy (World Health Organization, Meeting to develop a global consensus on preconception care to reduce maternal and childhood mortality and morbidity, 2013, Lassi et al., 2014). For example, a previous study by Wit et al. (Witt et al., 2012) found that women with poor mental health during the preconception period had higher risks of pregnancy complications, stillbirth and low birth weight. Previous studies also demonstrated the need for controlling already existing health conditions, such as pregestational diabetes or chronic hypertension, but these interventions target a limited number of women with identified risks. An extension of our model could integrate the longer-term effects of preconception care in children or elaborate on intermediate mechanisms acting on the different sub-systems. For example, the way in which adolescent pregnancy and early marriage are intrinsically linked, or the extent to which promoting schooling and reinforcing economic security contribute to reducing adolescent pregnancy rates, especially in the most disadvantaged communities (Chandra-Mouli et al., 2013, McQueston et al., 2013, Hindin et al., 2016). To create a CLD, it is important to establish limits and determine the level of analysis that will make it possible to reconcile details and overall balance. This implies making arbitrary decisions regarding the number and the nature of the constructs included in the model.

A few other limitations need to be considered in this study. Firstly, we only searched for studies written in English in PubMed and Embase, supplemented with hand searching on Google and Google Scholar, which may limit the number of included studies. Secondly, the *meta*-analyses that formed the basis of our analysis included studies conducted in various settings. This allowed us to incorporate a global perspective into the construction of the CLD and capture a comprehensive understanding of the pathways from preconception care to maternal and child mortality. However, since the effectiveness and implementation of preconception care interventions can be influenced by numerous contextual factors, it is important to acknowledge that the content of the CLD may not be applicable to all settings. Thirdly, using data from the literature is not a common methodology for creating a CLD. A more traditional way is to use primary data, generally obtained from participatory approaches, to identify the variables, build, and develop the CLD. However, this methodology has already been used in similar research. In a scoping review exploring the use of CLDs in public health research, Littlejohns et al. (Baugh Littlejohns et al., 2021) found that 10 out of 23 studies used secondary data as a main or complementary source of information. One of the complexities in constructing a CLD from the scientific literature is the treatment of non-significant associations. If these associations are valuable, they are impossible to incorporate into a CLD because they cannot be materialised. As such, our scoping review did not include non-significant associations, as their inclusion would not contribute meaningful information to the construction of the CLD. It is important to note that our decision to exclude non-significant associations does introduce a limitation in representing the full spectrum of evidence available. Another consequence of this approach is that the results we extracted did not reveal feedback loops, an important component in CLDs. Instead, the connections drawn from the *meta*-analyses were essentially linear. Again, working at the sub-system level and diversifying the data sources (i.e. interviews, expert workshops, group model building) would be a valuable approach to further investigating the complex mechanisms underlying preconception care and health.

### 5.1 Implications

The CLD provides a visualisation tool that stakeholders can use to rapidly obtain an overview of the different preconception pathways influencing maternal and child health and identify potential levers for action. It may also be a gateway for further research investigating how multiple interventions may act in synergy and produce cumulative effects. By narrowing the scope of the analysis and creating CLDs that focus specifically on one or two sub-systems, further research will obtain a more in-depth picture that could help identify specific mechanisms and feedback loops.

## 6 Conclusion

This study gathered evidence from 34 *meta*-analyses to offer an original perspective on preconception health and care. Using a CLD enabled us to describe the interplay of pathways linking preconception interventions to maternal, perinatal and child mortality. The CLD shows the potential of comprehensive strategies addressing multiple risk factors simultaneously and provides a tool to advocate for better integration of preconception care within strategies to prevent maternal and child mortality. This study constitutes a starting point for developing a preconception care investment case methodology. Our model lays interesting foundations while highlighting several limitations and areas for improvement. The lack of robust evidence on the effectiveness of some key interventions, such as preconception counselling, is a challenge for building a relevant and functional quantitative model, but this could be overcome by modifying the scope of our analysis and exploring other data sources.

## Funding

This research did not receive any specific grant from funding agencies in the public, commercial, or not-for-profit sectors.

## Declaration of Competing Interest

The authors declare that they have no known competing financial interests or personal relationships that could have appeared to influence the work reported in this paper.

## Data Availability

Data will be made available on request.

## References

[b0005] Arksey H., O'Malley L. (2005). Scoping studies: towards a methodological framework. Int. J. Soc. Res. Methodol..

[b0010] Atrash H.K., Johnson K., Adams M., Cordero J.F., Howse J. (2006). Preconception care for improving perinatal outcomes: the time to act. Matern. Child Health J..

[b0015] Aune D., Saugstad O.D., Henriksen T., Tonstad S. (2014). Physical activity and the risk of preeclampsia: a systematic review and meta-analysis. Epidemiology.

[b0020] Aune D., Sen A., Henriksen T., Saugstad O.D., Tonstad S. (2016). Physical activity and the risk of gestational diabetes mellitus: a systematic review and dose-response meta-analysis of epidemiological studies. Eur. J. Epidemiol..

[b0025] Baugh Littlejohns, L., C. Hill, and C. Neudorf, *Diverse Approaches to Creating and Using Causal Loop Diagrams in Public Health Research: Recommendations From a Scoping Review.* Public Health Rev, 2021. 42: p. 1604352-1604352. DOI: 10.3389/phrs.2021.1604352. 10.3389/phrs.2021.1604352 PMC8712315 35140995

[b0030] Berglund A., Lindmark G. (2016). Preconception health and care (PHC)—a strategy for improved maternal and child health. Ups. J. Med. Sci..

[b0035] Bhutta Z, D.S., Imam A, Lassi Z, *A Systematic Review of Preconception Risks and Interventions.* . 2011, The Aga Khan University.

[b0040] Bodnar, L.M., et al., *Periconceptional multivitamin use reduces the risk of preeclampsia.* Am J Epidemiol, 2006. **164**(5): p. 470-7. DOI: 10.1093/aje/kwj218. 10.1093/aje/kwj218 16772374

[b0045] Cao G., Liu J., Liu M. (2022). Global, Regional, and National Incidence and Mortality of Neonatal Preterm Birth, 1990–2019. JAMA Pediatr..

[b0050] Chandra-Mouli V., Camacho A.V., Michaud P.-A. (2013). WHO guidelines on preventing early pregnancy and poor reproductive outcomes among adolescents in developing countries. J. Adolesc. Health.

[b0055] Chin H.B., Sipe T.A., Elder R., Mercer S.L., Chattopadhyay S.K., Jacob V., Wethington H.R., Kirby D., Elliston D.B., Griffith M., Chuke S.O., Briss S.C., Ericksen I., Galbraith J.S., Herbst J.H., Johnson R.L., Kraft J.M., Noar S.M., Romero L.M., Santelli J. (2012). The effectiveness of group-based comprehensive risk-reduction and abstinence education interventions to prevent or reduce the risk of adolescent pregnancy, human immunodeficiency virus, and sexually transmitted infections: two systematic reviews for the Guide to Community Preventive Services. Am. J. Prev. Med..

[b0060] Conde-Agudelo A., Rosas-Bermúdez A., Kafury-Goeta A.C. (2006). Birth spacing and risk of adverse perinatal outcomes: a meta-analysis. J. Am. Med. Assoc..

[b0065] Coonrod, D.V., et al., *The clinical content of preconception care: immunizations as part of preconception care.* Am J Obstet Gynecol, 2008. **199**(6 Suppl 2): p. S290-5. DOI: 10.1016/j.ajog.2008.08.061. 10.1016/j.ajog.2008.08.061 19081423

[b0070] Damtie Y., Kefale B., Yalew M., Arefaynie M., Adane B., Haider M.R. (2021). Short birth spacing and its association with maternal educational status, contraceptive use, and duration of breastfeeding in Ethiopia. A systematic review and meta-analysis. PLoS One.

[b0075] Dean S.V., Lassi Z.S., Imam A.M., Bhutta Z.A. (2014). Preconception care: promoting reproductive planning. Reprod. Health.

[b0080] Dean S.V., Lassi Z.S., Imam A.M., Bhutta Z.A. (2014). Preconception care: nutritional risks and interventions. Reprod. Health.

[b0085] De-Regil L.M., Peña-Rosas J.P., Fernández-Gaxiola A.C., Rayco-Solon P. (2015). Effects and safety of periconceptional oral folate supplementation for preventing birth defects. Cochrane Database Syst. Rev..

[b0090] De-Regil, L.M., et al., *Effects and safety of periconceptional folate supplementation for preventing birth defects.* Cochrane Database Syst Rev, 2010(10): p. Cd007950. DOI: 10.1002/14651858.CD007950.pub2. 10.1002/14651858.CD007950.pub2 PMC4160020 20927767

[b0095] Dorney E., Black K.I. (2018). Preconception care. Aust. J. Gen. Pract..

[b0100] Elixhauser A., Weschler J.M., Kitzmiller J.L., Marks J.S., Bennert H.W., Coustan D.R., Gabbe S.G., Herman W.H., Kaufmann R.C., Ogata E.S., Sepe S.J. (1993). Cost-benefit analysis of preconception care for women with established diabetes mellitus. Diabetes Care.

[b0105] Flores A.L. (2014). Global burden of neural tube defects, risk factors, and prevention. Indian J. Community Health.

[b0110] Goossens J., De Roose M., Van Hecke A., Goemaes R., Verhaeghe S., Beeckman D. (2018). Barriers and facilitators to the provision of preconception care by healthcare providers: A systematic review. Int. J. Nurs. Stud..

[b0115] Grønvik T., Fossgard Sandøy I., van Wouwe J.P. (2018). Complications associated with adolescent childbearing in Sub-Saharan Africa: A systematic literature review and meta-analysis. PLoS One.

[b0120] Grosse S.D., Sotnikov S.V., Leatherman S., Curtis M. (2006). The business case for preconception care: methods and issues. Matern. Child Health J..

[b0125] Harden, A., et al., *Teenage pregnancy and social disadvantage: systematic review integrating controlled trials and qualitative studies.* BMJ, 2009. **339**: p. b4254. DOI: 10.1136/bmj.b4254. 10.1136/bmj.b4254 PMC2776931 19910400

[b0130] He X.J., Dai R.X., Hu C.L. (2020). Maternal prepregnancy overweight and obesity and the risk of preeclampsia: A meta-analysis of cohort studies. Obes. Res. Clin. Pract..

[b0135] Hemsing N., Greaves L., Poole N. (2017). Preconception health care interventions: A scoping review. Sex. Reprod. Healthc..

[b0140] Hindin M.J., Kalamar A.M., Thompson T.-A., Upadhyay U.D. (2016). Interventions to prevent unintended and repeat pregnancy among young people in low- and middle-income countries: A systematic review of the published and gray literature. J. Adolesc. Health.

[b0145] Imdad A., Yakoob M.Y., Bhutta Z.A. (2011). The effect of folic acid, protein energy and multiple micronutrient supplements in pregnancy on stillbirths. BMC Public Health.

[b0150] Keats E.C., Neufeld L.M., Garrett G.S., Mbuya M.N.N., Bhutta Z.A. (2019). Improved micronutrient status and health outcomes in low- and middle-income countries following large-scale fortification: evidence from a systematic review and meta-analysis. Am. J. Clin. Nutr..

[b0155] Korenbrot C.C. (2002). Preconception care: a systematic review. Matern. Child Health J..

[b0160] Kotirum S., Kiatpongsan S., Kapol N. (2021). Systematic review of economic evaluation studies on preconception care interventions. Health Care Women Int..

[b0165] Kozuki N., Lee A.CC., Silveira M.F., Sania A., Vogel J.P., Adair L., Barros F., Caulfield L.E., Christian P., Fawzi W., Humphrey J., Huybregts L., Mongkolchati A., Ntozini R., Osrin D., Roberfroid D., Tielsch J., Vaidya A., Black R.E., Katz J. (2013). The associations of parity and maternal age with small-for-gestational-age, preterm, and neonatal and infant mortality: a meta-analysis. BMC Public Health.

[b0170] Kozuki N., Lee A.CC., Silveira M.F., Victora C.G., Adair L., Humphrey J., Ntozini R., Black R.E., Katz J. (2013). The associations of birth intervals with small-for-gestational-age, preterm, and neonatal and infant mortality: a meta-analysis. BMC Public Health.

[b0175] Laelago T., Yohannes T., Tsige G. (2020). Determinants of preterm birth among mothers who gave birth in East Africa: systematic review and meta-analysis. Ital. J. Pediatr..

[b0180] Lassi Z.S., Mansoor T., Salam R.A., Das J.K., Bhutta Z.A. (2014). Essential pre-pregnancy and pregnancy interventions for improved maternal, newborn and child health. Reprod. Health.

[b0185] Lassi Z.S., Imam A.M., Dean S.V., Bhutta Z.A. (2014). Preconception care: caffeine, smoking, alcohol, drugs and other environmental chemical/radiation exposure. Reprod. Health.

[b0190] Lassi Z.S., Dean S.V., Mallick D., Bhutta Z.A. (2014). Preconception care: delivery strategies and packages for care. Reprod. Health.

[b0195] Lassi Z.S., Kedzior S.G.E., Tariq W., Jadoon Y., Das J.K., Bhutta Z.A. (2020). Effects of preconception care and periconception interventions on maternal nutritional status and birth outcomes in low- and middle-income countries: a systematic review. Nutrients.

[b0200] Li B., Zhang X., Peng X., Zhang S., Wang X., Zhu C. (2019). Folic acid and risk of preterm birth: a meta-analysis. Front. Neurosci..

[b0205] Lin G., Palopoli M., Dadwal V., Celi L.A., Majumder M.S., Ordóñez P., Osorio J.S., Paik K.E., Somai M. (2020). Leveraging Data Science for Global Health.

[b0210] Liu L., Ma Y., Wang N., Lin W., Liu Y., Wen D. (2019). Maternal body mass index and risk of neonatal adverse outcomes in China: a systematic review and meta-analysis. BMC Pregn. Childbirth.

[b0215] Liu P., Xu L., Wang Y., Zhang Y., Du Y., Sun Y., Wang Z. (2016). Association between perinatal outcomes and maternal pre-pregnancy body mass index. Obes. Rev..

[b0220] M’hamdi H.I., van Voorst S.F., Pinxten W., Hilhorst M.T., Steegers E.A.P. (2017). Barriers in the uptake and delivery of preconception care: Exploring the views of care providers. Matern. Child Health J..

[b0225] Marvin-Dowle K., Soltani H. (2020). A comparison of neonatal outcomes between adolescent and adult mothers in developed countries: a systematic review and meta-analysis. Eur. J. Obstet. Gynecol. Reprod. Biol. X.

[b0230] Mason E., Chandra-Mouli V., Baltag V., Christiansen C., Lassi Z.S., Bhutta Z.A. (2014). Preconception care: advancing from 'important to do and can be done' to 'is being done and is making a difference'. Reprod. Health.

[b0235] Mason-Jones A.J., Sinclair D., Mathews C., Kagee A., Hillman A., Lombard C. (2016). School-based interventions for preventing HIV, sexually transmitted infections, and pregnancy in adolescents. Cochr. Database Syst. Rev..

[b0240] Mazza D., Chapman A., Michie S. (2013). Barriers to the implementation of preconception care guidelines as perceived by general practitioners: a qualitative study. BMC Health Serv. Res..

[b0245] McQueston K., Silverman R., Glassman A. (2013). The efficacy of interventions to reduce adolescent childbearing in low- and middle-income countries: a systematic review. Stud. Fam. Plann..

[b0250] Mijatovic-Vukas J., Capling L., Cheng S., Stamatakis E., Louie J., Cheung N., Markovic T., Ross G., Senior A., Brand-Miller J., Flood V. (2018). Associations of diet and physical activity with risk for gestational diabetes mellitus: a systematic review and meta-analysis. Nutrients.

[b0255] Najafi F., Hasani J., Izadi N., Hashemi‐Nazari S.-S., Namvar Z., Mohammadi S., Sadeghi M. (2019). The effect of prepregnancy body mass index on the risk of gestational diabetes mellitus: a systematic review and dose-response meta-analysis. Obes. Rev..

[b0260] Nesari M., Olson J.K., Vandermeer B., Slater L., Olson D.M. (2018). Does a maternal history of abuse before pregnancy affect pregnancy outcomes? A systematic review with meta-analysis. BMC Pregn. Childbirth.

[b0265] Putra I.C.S., Irianto C.B., Raffaello W.M., Suciadi L.P., Prameswari H.S. (2022). Pre-pregnancy obesity and the risk of peripartum cardiomyopathy: a systematic review and meta-analysis. Indian Heart J..

[b0270] Raghuraman N., Tuuli M.G. (2021). Preconception care as an opportunity to optimize pregnancy outcomes. J. Am. Med. Assoc..

[b0275] Rodrigues V.B., Silva E.N.d., Santos M.L.P., Wieringa F. (2021). Cost-effectiveness of mandatory folic acid fortification of flours in prevention of neural tube defects: a systematic review. PLoS One.

[b0280] Say, L., et al., *Global causes of maternal death: a WHO systematic analysis.* Lancet Glob Health, 2014. **2**(6): p. e323-33. DOI: 10.1016/s2214-109x(14)70227-x. 10.1016/S2214-109X(14)70227-X 25103301

[b0285] Tricco A.C., Lillie E., Zarin W., O'Brien K.K., Colquhoun H., Levac D., Moher D., Peters M.D.J., Horsley T., Weeks L., Hempel S., Akl E.A., Chang C., McGowan J., Stewart L., Hartling L., Aldcroft A., Wilson M.G., Garritty C., Lewin S., Godfrey C.M., Macdonald M.T., Langlois E.V., Soares-Weiser K., Moriarty J.o., Clifford T., Tunçalp Ö., Straus S.E. (2018). PRISMA extension for scoping reviews (PRISMA-ScR): checklist and explanation. Ann. Intern. Med..

[b0290] Ulhaq RenataAlya, Anis W., Fatmaningrum W., Akbar M.A. (2021). Association between pre-pregnancy body mass index and gestational weight gain and the risk of preeclampsia: a systematic review and meta-analysis. Asian Pacific J. Reprod..

[b0295] US Agency for International Development, *Local Systems: A framework for supporting sustained development*. 2014. p. 23.

[b0300] Vats H., Saxena R., Sachdeva M.P., Walia G.K., Gupta V. (2021). Impact of maternal pre-pregnancy body mass index on maternal, fetal and neonatal adverse outcomes in the worldwide populations: a systematic review and meta-analysis. Obes. Res. Clin. Pract..

[b0305] Walani S.R. (2020). Global burden of preterm birth. Int. J. Gynaecol. Obstet..

[b0310] Whitworth, M. and T. Dowswell, *Routine pre-pregnancy health promotion for improving pregnancy outcomes.* Cochrane Database Syst Rev, 2009(4): p. Cd007536. DOI: 10.1002/14651858.CD007536.pub2. 10.1002/14651858.CD007536.pub2 PMC4164828 19821424

[b0315] Wilson R.D., O'Connor D.L., No G. (2022). 427: folic acid and multivitamin supplementation for prevention of folic acid-sensitive congenital anomalies. J. Obstet. Gynaecol. Can..

[b0320] Witt W.P., Wisk L.E., Cheng E.R., Hampton J.M., Hagen E.W. (2012). Preconception mental health predicts pregnancy complications and adverse birth outcomes: a national population-based study. Matern. Child Health J..

[b0325] World Health Organization. *Maternal Mortality*. 2019 [11/03/2022]; Available from: https://www.who.int/news-room/fact-sheets/detail/maternal-mortality.

[b0330] World Health Organization, *Meeting to develop a global consensus on preconception care to reduce maternal and childhood mortality and morbidity*. 2013, World Health Organization,: Geneva.

[b0335] World Health Organization. *Newborn Mortality*. 2022 [11/03/2022]; Available from: https://www.who.int/news-room/fact-sheets/detail/levels-and-trends-in-child-mortality-report-2021.

[b0340] Yang Y., Cai Z., Zhang J. (2021). The effect of prepregnancy body mass index on maternal micronutrient status: a meta-analysis. Sci. Rep..

[b0345] Ye Z., Wang L., Yang T., Chen L., Wang T., Chen L., Zhao L., Zhang S., Zheng Z., Luo L., Qin J. (2019). Maternal viral infection and risk of fetal congenital heart diseases: a meta-analysis of observational studies. J. Am. Heart Assoc..

[b0350] Yu Z., Han S., Zhu J., Sun X., Ji C., Guo X., Baradaran H.R. (2013). Pre-pregnancy body mass index in relation to infant birth weight and offspring overweight/obesity: a systematic review and meta-analysis. PLoS One.

[b0355] Zhang Q., Wang Y., Xin X., Zhang Y.a., Liu D., Peng Z., He Y., Xu J., Ma X.u. (2017). Effect of folic acid supplementation on preterm delivery and small for gestational age births: a systematic review and meta-analysis. Reprod. Toxicol..

[b0360] Zhang Y.u., Xiao C.-M., Zhang Y., Chen Q., Zhang X.-Q., Li X.-F., Shao R.-Y., Gao Y.-M., Sugawara A. (2021). Factors associated with gestational diabetes mellitus: a meta-analysis. J. Diabetes Res..

