# Supplementary material for: Investigating the pathways from preconception care to preventing maternal, perinatal and child mortality: A scoping review and causal loop diagram
Source: Prev Med Rep. 2023 Jun 5;34:102274. doi: 10.1016/j.pmedr.2023.102274 (PMC10302151; doi:10.1016/j.pmedr.2023.102274)
Supplement: Supplementary data 1 [file mmc1.docx]

**Multiple Search Strategy**

1. **Outcomes**
   1. Teenage Pregnancy

| **Database** | **Syntax** |
| --- | --- |
| **Pubmed** | (("adolescent pregnancy"[tiab] OR "teenage pregnancy"[tiab] OR "early pregnancy"[tiab]) AND ("outcome"[tiab] OR "outcome"[tiab] OR "effects"[tiab] OR "effect"[tiab] OR "complications"[tiab])) AND ((meta-analysis[Filter]) AND (2002:2022[pdat])) |
| **Embase** | ('adolescent pregnancy':ab,ti OR 'teenage pregnancy':ab,ti OR 'early pregnancy':ab,ti) AND ('outcomes':ab,ti OR 'outcome':ab,ti OR 'effect':ab,ti OR 'effects':ab,ti OR 'complications':ab,ti) AND [meta analysis]/lim AND [2002-2022]/py |

- 1. Birth Spacing

| **Database** | **Syntax** |
| --- | --- |
| **Pubmed** | (("birth interval"[tiab] OR "birth spacing"[tiab] OR "pregnancy interval"[tiab]) AND ("outcome"[tiab] OR "outcome"[tiab] OR "effects"[tiab] OR "effect"[tiab] OR "complications"[tiab])) AND ((meta-analysis[Filter]) AND (2002:2022[pdat])) |
| **Embase** | ('birth spacing':ab,ti OR 'birth interval':ab,ti OR 'pregnancy interval':ab,ti) AND ('outcomes':ab,ti OR 'outcome':ab,ti OR 'effect':ab,ti OR 'effects':ab,ti OR 'complications':ab,ti) AND [meta analysis]/lim AND [2002-2022]/py |

- 1. Micronutrients Deficiency

| **Database** | **Syntax** |
| --- | --- |
| **Pubmed** | (("preconception"[Title/Abstract] OR "prepregnancy"[Title/Abstract] OR "pre-pregnancy"[Title/Abstract] OR "before conception"[All Fields] OR "before pregnancy"[Title/Abstract] OR "preconception"[Title/Abstract]) AND ("iron"[Title/Abstract] OR "folic acid"[Title/Abstract] OR "folate"[Title/Abstract] OR "micronutrients"[Title/Abstract]) AND ("outcomes"[Title/Abstract] OR "outcome"[Title/Abstract] OR "effects"[Title/Abstract] OR "effect"[Title/Abstract] OR "complications"[Title/Abstract])) AND (meta-analysis[Filter]) AND (2002:2022[pdat])) |
| **Embase** | ('preconception':ab,ti OR 'pre pregnancy':ab,ti OR 'pre-pregnancy':ab,ti OR 'before pregnancy':ab,ti OR 'before conception':ab,ti) AND ('iron':ab,ti OR 'folic acid':ab,ti OR 'folate':ab,ti OR 'micronutrients':ab,ti) AND ('outcomes':ab,ti OR 'outcome':ab,ti OR 'effect':ab,ti OR 'effects':ab,ti OR 'complications':ab,ti) AND [meta analysis]/lim AND [2002-2022]/py |

- 1. Pre-Pregnancy Weight

| **Database** | **Syntax** |
| --- | --- |
| **Pubmed** | (("preconception"[Title/Abstract] OR "prepregnancy"[Title/Abstract] OR "pre-pregnancy"[Title/Abstract] OR "before conception"[All Fields] OR "before pregnancy"[Title/Abstract] OR "preconception"[Title/Abstract]) AND ("overweight"[Title/Abstract] OR "underweight"[Title/Abstract] OR "BMI"[Title/Abstract] OR "body mass index"[Title/Abstract] OR "nutritional status"[Title/Abstract] OR "obesity"[Title/Abstract] OR "obese"[Title/Abstract] OR "weight"[Title/Abstract]) AND ("outcomes"[Title/Abstract] OR "outcome"[Title/Abstract] OR "effects"[Title/Abstract] OR "effect"[Title/Abstract] OR "complications"[Title/Abstract])) AND (meta-analysis[Filter]) AND (2002:2022[pdat])) |
| **Embase** | ('preconception':ab,ti OR 'pre pregnancy':ab,ti OR 'pre-pregnancy':ab,ti OR 'before pregnancy':ab,ti OR 'before conception':ab,ti) AND ('overweight':ab,ti OR 'underweight':ab,ti OR 'bmi':ab,ti OR 'body mass index':ab,ti OR 'nutritional status':ab,ti OR 'obesity':ab,ti OR 'obese':ab,ti OR 'weight':ab,ti) AND ('outcomes':ab,ti OR 'outcome':ab,ti OR 'effect':ab,ti OR 'effects':ab,ti OR 'complications':ab,ti) AND [meta analysis]/lim AND [2002-2022]/py |

- 1. Smoking and Alcohol

| **Database** | **Syntax** |
| --- | --- |
| **Pubmed** | ("preconception"[tiab] OR "prepregnancy"[tiab] OR "pre-pregnancy"[tiab] OR "before conception" OR "before pregnancy"[tiab] OR "preconception"[tiab]) AND ("smoking"[tiab] OR "tobacco"[tiab] OR "alcohol"[tiab]) AND ("outcomes"[tiab] OR "outcome"[tiab] OR "effects"[tiab] OR "effect"[tiab] OR "complications"[tiab]) AND (meta-analysis[Filter]) AND (2002:2022[pdat])) |
| **Embase** | ('preconception':ab,ti OR 'pre pregnancy':ab,ti OR 'pre-pregnancy':ab,ti OR 'before pregnancy':ab,ti OR 'before conception':ab,ti) AND ('smoking':ab,ti OR 'tobacco':ab,ti OR 'alcohol':ab,ti) AND ('outcomes':ab,ti OR 'outcome':ab,ti OR 'effect':ab,ti OR 'effects':ab,ti OR 'complications':ab,ti) AND [meta analysis]/lim AND [2002-2022]/py |

- 1. Vaccine-Preventable Diseases

| **Database** | **Syntax** |
| --- | --- |
| **Pubmed** | ("preconception"[tiab] OR "prepregnancy"[tiab] OR "pre-pregnancy"[tiab] OR "before conception" OR "before pregnancy"[tiab] OR "preconception"[tiab]) AND ("vaccine"[tiab] OR "vaccines"[tiab] OR "immunisation"[tiab] OR "rubella"[tiab] OR "measles"[tiab] OR "varicella"[tiab]) AND ("outcomes"[tiab] OR "outcome"[tiab] OR "effects"[tiab] OR "effect"[tiab] OR "complications"[tiab]) AND (meta-analysis[Filter]) AND (2002:2022[pdat])) |
| **Embase** | ('preconception':ab,ti OR 'pre pregnancy':ab,ti OR 'pre-pregnancy':ab,ti OR 'before pregnancy':ab,ti OR 'before conception':ab,ti) AND ('vaccine':ab,ti OR 'immunisation':ab,ti OR 'rubella':ab,ti OR 'measles':ab,ti OR 'varicella':ab,ti) AND ('outcomes':ab,ti OR 'outcome':ab,ti OR 'effect':ab,ti OR 'effects':ab,ti OR 'complications':ab,ti) AND [meta analysis]/lim AND [2002-2022]/py |

- 1. Violence Against Women

| **Database** | **Syntax** |
| --- | --- |
| **Pubmed** | ("preconception"[tiab] OR "prepregnancy"[tiab] OR "pre-pregnancy"[tiab] OR "before conception" OR "before pregnancy"[tiab] OR "preconception"[tiab]) AND ("violence"[tiab] OR "domestic violence"[tiab] OR "intimate partner violence"[tiab] OR "violence against women"[tiab] OR "gender-based violence"[tiab]) AND ("outcomes"[tiab] OR "outcome"[tiab] OR "effects"[tiab] OR "effect"[tiab] OR "complications"[tiab]) AND (meta-analysis[Filter]) AND (2002:2022[pdat])) |
| **Embase** | ('preconception':ab,ti OR 'pre pregnancy':ab,ti OR 'pre-pregnancy':ab,ti OR 'before pregnancy':ab,ti OR 'before conception':ab,ti) AND ('violence':ab,ti OR 'domestic violence':ab,ti OR 'gender based violence':ab,ti OR 'intimate partner violence':ab,ti OR 'ipv':ab,ti) AND ('outcomes':ab,ti OR 'outcome':ab,ti OR 'effect':ab,ti OR 'effects':ab,ti OR 'complications':ab,ti) AND [meta analysis]/lim AND [2002-2022]/py |

1. **Interventions**
   1. Teenage Pregnancy

| **Database** | **Syntax** |
| --- | --- |
| **Pubmed** | (("adolescent pregnancy"[tiab] OR "teenage pregnancy"[tiab] OR "early pregnancy"[tiab]) AND ("intervention"[tiab] OR "interventions"[tiab] OR "policy"[tiab] OR "policies"[tiab] OR "impact"[tiab] OR "impacts"[tiab] OR "program"[tiab] OR "programme"[tiab])) AND ((meta-analysis[Filter]) AND (meta-analysis[Filter]) AND (2002:2022[pdat])) |
| **Embase** | ('adolescent pregnancy':ab,ti OR 'teenage pregnancy':ab,ti OR 'early pregnancy':ab,ti) AND ('intervention':ab,ti OR 'interventions':ab,ti OR 'policy':ab,ti OR 'policies':ab,ti OR 'impact':ab,ti OR 'impacts':ab,ti OR 'program':ab,ti OR 'programme':ab,ti) AND [meta analysis]/lim AND [2002-2022]/py |

- 1. Birth Spacing

| **Database** | **Syntax** |
| --- | --- |
| **Pubmed** | (("birth interval"[tiab] OR "birth spacing"[tiab] OR "pregnancy interval"[tiab]) AND ("intervention"[tiab] OR "interventions"[tiab] OR "policy"[tiab] OR "policies"[tiab] OR "impact"[tiab] OR "impacts"[tiab] OR "program"[tiab] OR "programme"[tiab])) AND ((meta-analysis[Filter]) AND (meta-analysis[Filter]) AND (2002:2022[pdat])) |
| **Embase** | ('birth spacing':ab,ti OR 'birth interval':ab,ti OR 'pregnancy interval':ab,ti) AND ('intervention':ab,ti OR 'interventions':ab,ti OR 'policy':ab,ti OR 'policies':ab,ti OR 'impact':ab,ti OR 'impacts':ab,ti OR 'program':ab,ti OR 'programme':ab,ti) AND [meta analysis]/lim AND [2002-2022]/py |

- 1. Micronutrients Deficiency

| **Database** | **Syntax** |
| --- | --- |
| **Pubmed** | (("preconception"[Title/Abstract] OR "prepregnancy"[Title/Abstract] OR "pre-pregnancy"[Title/Abstract] OR "before conception"[All Fields] OR "before pregnancy"[Title/Abstract] OR "preconception"[Title/Abstract]) AND ("intervention"[tiab] OR "interventions"[tiab] OR "policy"[tiab] OR "policies"[tiab] OR "impact"[tiab] OR "impacts"[tiab] OR "program"[tiab] OR "programme"[tiab])) AND (meta-analysis[Filter]) AND (2002:2022[pdat])) |
| **Embase** | ('preconception':ab,ti OR 'pre pregnancy':ab,ti OR 'pre-pregnancy':ab,ti OR 'before pregnancy':ab,ti OR 'before conception':ab,ti) AND ('iron':ab,ti OR 'folic acid':ab,ti OR 'folate':ab,ti OR 'micronutrients':ab,ti) AND ('intervention':ab,ti OR 'interventions':ab,ti OR 'policy':ab,ti OR 'policies':ab,ti OR 'impact':ab,ti OR 'impacts':ab,ti OR 'program':ab,ti OR 'programme':ab,ti) AND [meta analysis]/lim AND [2002-2022]/py |

- 1. Pre-Pregnancy Weight

| **Database** | **Syntax** |
| --- | --- |
| **Pubmed** | (("preconception"[Title/Abstract] OR "prepregnancy"[Title/Abstract] OR "pre-pregnancy"[Title/Abstract] OR "before conception"[All Fields] OR "before pregnancy"[Title/Abstract] OR "preconception"[Title/Abstract]) AND ("overweight"[Title/Abstract] OR "underweight"[Title/Abstract] OR "BMI"[Title/Abstract] OR "body mass index"[Title/Abstract] OR "nutritional status"[Title/Abstract] OR "obesity"[Title/Abstract] OR "obese"[Title/Abstract] OR "weight"[Title/Abstract]) AND ("intervention"[tiab] OR "interventions"[tiab] OR "policy"[tiab] OR "policies"[tiab] OR "impact"[tiab] OR "impacts"[tiab] OR "program"[tiab] OR "programme"[tiab])) AND ((meta-analysis[Filter]) AND (2002:2022[pdat])) |
| **Embase** | ('preconception':ab,ti OR 'pre pregnancy':ab,ti OR 'pre-pregnancy':ab,ti OR 'before pregnancy':ab,ti OR 'before conception':ab,ti) AND ('overweight':ab,ti OR 'underweight':ab,ti OR 'bmi':ab,ti OR 'body mass index':ab,ti OR 'nutritional status':ab,ti OR 'obesity':ab,ti OR 'obese':ab,ti OR 'weight':ab,ti) AND ('intervention':ab,ti OR 'interventions':ab,ti OR 'policy':ab,ti OR 'policies':ab,ti OR 'impact':ab,ti OR 'impacts':ab,ti OR 'program':ab,ti OR 'programme':ab,ti) AND [meta analysis]/lim AND [2002-2022]/py |

- 1. Smoking and Alcohol

| **Database** | **Syntax** |
| --- | --- |
| **Pubmed** | ("preconception"[tiab] OR "prepregnancy"[tiab] OR "pre-pregnancy"[tiab] OR "before conception" OR "before pregnancy"[tiab] OR "preconception"[tiab]) AND ("smoking"[tiab] OR "tobacco"[tiab] OR "alcohol"[tiab]) AND ("intervention"[tiab] OR "interventions"[tiab] OR "policy"[tiab] OR "policies"[tiab] OR "impact"[tiab] OR "impacts"[tiab] OR "program"[tiab] OR "programme"[tiab])) AND ((meta-analysis[Filter]) AND (2002:2022[pdat])) |
| **Embase** | ('preconception':ab,ti OR 'pre pregnancy':ab,ti OR 'pre-pregnancy':ab,ti OR 'before pregnancy':ab,ti OR 'before conception':ab,ti) AND ('smoking':ab,ti OR 'tobacco':ab,ti OR 'alcohol':ab,ti) AND ('intervention':ab,ti OR 'interventions':ab,ti OR 'policy':ab,ti OR 'policies':ab,ti OR 'impact':ab,ti OR 'impacts':ab,ti OR 'program':ab,ti OR 'programme':ab,ti) AND [meta analysis]/lim AND [2002-2022]/py |

- 1. Vaccine-Preventable Diseases

| **Database** | **Syntax** |
| --- | --- |
| **Pubmed** | ("preconception"[tiab] OR "prepregnancy"[tiab] OR "pre-pregnancy"[tiab] OR "before conception" OR "before pregnancy"[tiab] OR "preconception"[tiab]) AND ("vaccine"[tiab] OR "vaccines"[tiab] OR "immunisation"[tiab] OR "rubella"[tiab] OR "measles"[tiab] OR "varicella"[tiab]) AND ("intervention"[tiab] OR "interventions"[tiab] OR "policy"[tiab] OR "policies"[tiab] OR "impact"[tiab] OR "impacts"[tiab] OR "program"[tiab] OR "programme"[tiab])) AND ((meta-analysis[Filter]) AND (2002:2022[pdat])) |
| **Embase** | ('preconception':ab,ti OR 'pre pregnancy':ab,ti OR 'pre-pregnancy':ab,ti OR 'before pregnancy':ab,ti OR 'before conception':ab,ti) AND ('vaccine':ab,ti OR 'immunisation':ab,ti OR 'rubella':ab,ti OR 'measles':ab,ti OR 'varicella':ab,ti) AND ('intervention':ab,ti OR 'interventions':ab,ti OR 'policy':ab,ti OR 'policies':ab,ti OR 'impact':ab,ti OR 'impacts':ab,ti OR 'program':ab,ti OR 'programme':ab,ti) AND [meta analysis]/lim AND [2002-2022]/py |

- 1. Violence Against Women

| **Database** | **Syntax** |
| --- | --- |
| **Pubmed** | ("preconception"[tiab] OR "prepregnancy"[tiab] OR "pre-pregnancy"[tiab] OR "before conception" OR "before pregnancy"[tiab] OR "preconception"[tiab]) AND ("violence"[tiab] OR "domestic violence"[tiab] OR "intimate partner violence"[tiab] OR "violence against women"[tiab] OR "gender-based violence"[tiab]) AND ("intervention"[tiab] OR "interventions"[tiab] OR "policy"[tiab] OR "policies"[tiab] OR "impact"[tiab] OR "impacts"[tiab] OR "program"[tiab] OR "programme"[tiab])) AND ((meta-analysis[Filter]) AND (2002:2022[pdat])) |
| **Embase** | ('preconception':ab,ti OR 'pre pregnancy':ab,ti OR 'pre-pregnancy':ab,ti OR 'before pregnancy':ab,ti OR 'before conception':ab,ti) AND ('violence':ab,ti OR 'domestic violence':ab,ti OR 'gender based violence':ab,ti OR 'intimate partner violence':ab,ti OR 'ipv':ab,ti) AND ('intervention':ab,ti OR 'interventions':ab,ti OR 'policy':ab,ti OR 'policies':ab,ti OR 'impact':ab,ti OR 'impacts':ab,ti OR 'program':ab,ti OR 'programme':ab,ti) AND [meta analysis]/lim AND [2002-2022]/py |
